# *Gentianella macrosperma*, a new species of *Gentianella* (Gentianaceae) from Xinjiang, China

**DOI:** 10.3897/phytokeys.130.35476

**Published:** 2019-08-29

**Authors:** Hai-Feng Cao, Ji-Dong Ya, Qiao-Rong Zhang, Xiao-Jian Hu, Zhi-Rong Zhang, Xin-Hua Liu, Yong-Cheng Zhang, Ai-Ting Zhang, Wen-Bin Yu

**Affiliations:** 1 Shanghai Museum of TCM, Shanghai University of Traditional Chinese Medicine, Shanghai 201203, China Shanghai University of Traditional Chinese Medicine Shanghai China; 2 Germplasm Bank of Wild Species, Kunming Institute of Botany, Chinese Academy of Sciences, Lanhei Road 132, Heilongtan, Kunming, Yunnan, 650201, China Kunming Institute of Botany, Chinese Academy of Sciences Kunming China; 3 Ili Botanical Garden, Xinjiang Institute of Ecology and Geography, Chinese Academy of Sciences, Xinyuan, Xinjiang, 835815, China Xinjiang Institute of Ecology and Geography, Chinese Academy of Sciences Xinyuan China; 4 Forestry Bureau of Xinyuan County, Xinyuan, Xinjiang, 835800, China Forestry Bureau of Xinyuan County Xinyuan China; 5 Xinjiang Agricultural Broadcasting and Television School, Xinyuan, Xinjiang, 835800, China Xinjiang Agricultural Broadcasting and Television School Xinyuan China; 6 Center for Integrative Conservation, Xishuangbanna Tropical Botanical Garden, Chinese Academy of Sciences, Mengla 666303, Yunnan, China Xishuangbanna Tropical Botanical Garden Mengla China; 7 Center of Conservation Biology, Core Botanical Gardens, Chinese Academy of Sciences, Mengla 666303, Yunnan, China Center of Conservation Biology, Core Botanical Gardens Mengla China

**Keywords:** *
Gentianella
*, ITS, matK, Morphology, Swertiinae, Taxonomy, Xinjiang

## Abstract

*Gentianella macrosperma* Ma ex H.F. Cao, J.D. Ya & Q.R. Zhang, a new species of Gentianaceae from Xinjiang, Northwest China is described and illustrated. This new species is unique in having equal length of corolla lobe and corolla tube, nectaries located at the throat of the corolla tube and large seeds up to 1.6 mm in diameter. In addition, an updated identification key to the Chinese species of *Gentianella* is provided.

## Introduction

*Gentianella* Moench (Gentianaceae) consists of approximately 300 species distributed from the temperate, arctic and alpine regions of the Northern Hemisphere, to South America, Australia and New Zealand (Pringle 2017). About 70% of species (ca. 200 species) occur in South America, where new species continue to be discovered (Pfanzelt et al. 2015; Pringle 2015, 2017; Pringle and Grant 2017). Molecular phylogenetic studies indicated that *Gentianella* was polyphyletic, and the new circumscription of *Gentianella* s. str. contains species with one nectary per petal lobe (von Hagen and Kadereit 2001, 2002). However, the taxonomic placement of the Asiatic species with two nectaries per corolla lobe has yet to be determined. Before the phylogenetically-based concept of Asiatic gentianellas proposed, the description of this genus published in Flora of China (Ho and Pringle 1995) remains applicable in the present context. There are 10 species of *Gentianella* reported from China and mainly distributed in northern China and alpine areas of southwest China mountains (Ho and Pringle 1995, Chen et al. 2011).

During the field expedition to west of Xinjiang, China, an unusual species of Gentianaceae was collected. Its corolla campanulate without plicae and fringed scale, lobed to middle of corolla, two nectaries per corolla lobe located at the corolla tube fit the main characters of *Gentianella*. Subsequent morphological investigation and molecular study supported this species as new to science and described here.

## Materials and methods

Specimen collections of *Gentianella* were carefully examined, especially the relevant species, including *G. holosteoides* Schott & Kotschy ex N.M. Pritch., *G. longicarpa* (Gilli) Holub, *G. sibirica* (Kusn.) Holub, *G. stoliczkae* (Kurz ex C.B. Clarke) Holub and *G. umbellata* (M. Bieb.) Holub. Collections at the following herbaria (BM, FR, GH, GLM, HIMC, HNWP, JE, K, E, KFTA, KUN, MA, MPU, MW, P, PE, PEY, W, WAG) were checked on-site and via Chinese Virtual Herbarium (CVH, http://www.cvh.ac.cn/), Global Biodiversity Information Facility (GBIF, https://www.gbif.org/) and Global Plants on JSTOR (https://plants.jstor.org/). The high-resolution images of type specimen of *G. sibirica* (LE01043410, LE01043411, LE00050650) were obtained from curators of LE. Relevant literatures were investigated (Gillett 1957; Shishkin and Bobrov 1967; Omer et al. 1988; Ho and Pringle 1995; Omer 1995; Struwe et al. 2002; Aitken 2007; Chen et al. 2011; Mohd et al. 2018). Line drawings, description and most of photographs were based on the latest collections (J.D. Ya et al. *17CS16327*), except that the images of seeds were from the type specimen (Shun-Li Chen *Tianyi281*, PE00029466). The conservation status of the new species was evaluated according to the guidelines of the IUCN Red List Categories and Criteria (IUCN 2017)

Fresh leaves of this new species were dried immediately by using silica gel for DNA extraction. Genomic DNA extraction, amplification and DNA sequencing of ITS and the plastid *matK* followed the protocol described by Xi et al. (2014) and sequences of relevant species were downloaded from GenBank (Appendix 1).

**Figure 1.**
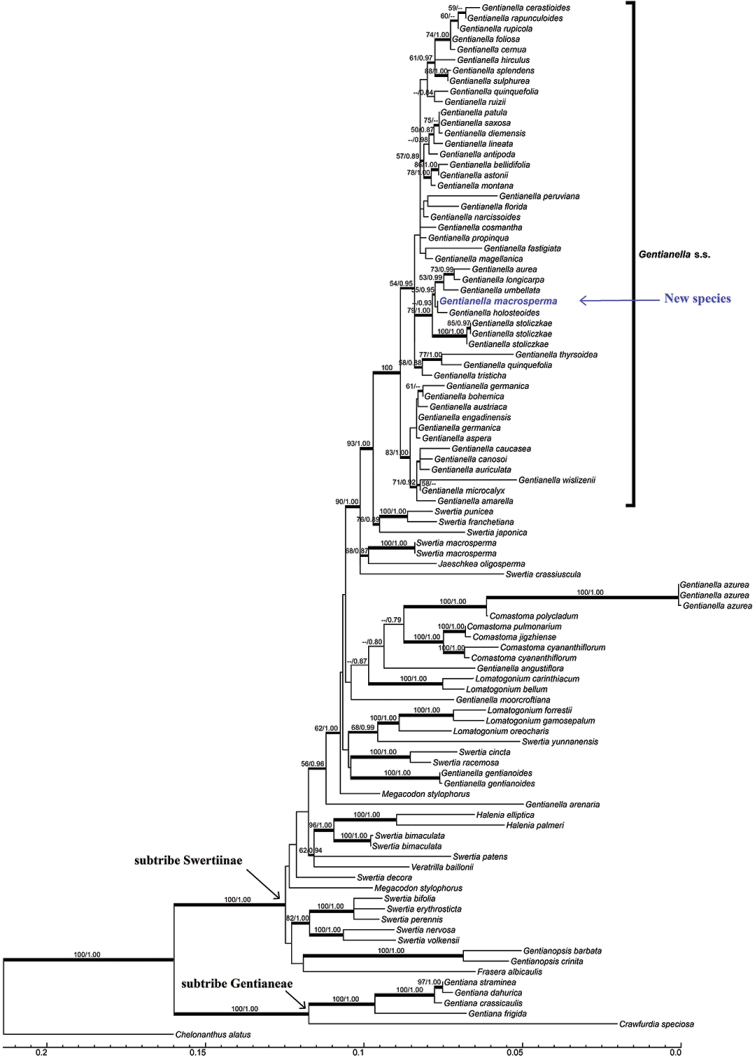
The major-rule consensus tree of ML analysis based on the total dataset, including ITS and *matK*. ML bootstrap values and BI posterior probabilities are shown on branches.

The molecular phylogenetic tree of 88 species representing 13 genera of Gentianaceae was reconstructed using Bayesian Inference (BI) and Maximum Likelihood (ML). *Chelonanthus alatus* (Aubl.) Pulle (Gentianaceae: Helieae) was chosen as outgroup (Figure 1). ITS and *matK* datasets were combined for analysis. BI analysis was performed using MrBayes 3.26 (Ronquist and Huelsenbeck 2003). Markov Chain Monte Carlo (MCMC) analysis was performed using MrBayes for 10,000,000 generations for the combined dataset, with two simultaneous runs, with each run comprising four incrementally heated chains. BI analysis was started with a random tree and sampled every 1000 generations. The combined dataset was partitioned and the best-fit DNA substitution model for two DNA regions using Bayesian Information Criterion (BIC) was estimated using jModeltest 2 (Darriba et al. 2012). ML analysis was conducted with RAxML 8.2.10 (Stamatakis et al. 2008) using the GTR substitution model with gamma-distributed rate heterogeneity amongst sites and the proportion of invariable sites estimated from the data. Support values for nodes/clades were estimated from 1000 bootstrap replicates.

## Results

The ITS matrix was 689 bp in length including 376 variable sites and 266 parsimony-informative sites and the *matK* matrix was 821 bp in length including 286 variable sites and 198 parsimony-informative sites. The best-fit BIC model of ITS and *matK* datasets was SYM+G and TVM+G, respectively. The major-rule consensus tree of both BI and ML analyses with support values is shown in Figure 1.

Phylogenetic analyses using ML and BI methods identified that *Gentianella*, *Swertia* L. and other genera in subtribe Swertiinae are not monophyletic, which shows a similar conclusion as previous studies (von Hagen and Kadereit 2001, 2002; Xi et al. 2014). Current new species and 44 other *Gentianella* species were strongly supported as monophyletic (BI PP = 1.00, ML BS = 93; Figure 1). *G. arenaria* (Maxim.) T.N. Ho, *G. angustiflora* H. Smith, *G. azurea* (Bunge) Holub, *G. gentianoides* (Franch.) H. Smith and *G. moorcroftiana* (Wall. ex G. Don) A. Shaw formed different clades with *Comastoma* Toyok., *Lomatogonium* A. Braun, *Swertia* and other genera in Swertiinae.

Phylogenetic analyses showed that this new species and *G. holosteoides* formed a clade (BI PP = 0.93), then sister to the clade including *G. aurea* (L.) H. Smith, *G. umbellata* and *G. longicarpa* (Figure 1). Three samples of *G. stoliczkae* were located at most basal of the new species clade (BI PP = 1.00, ML BS = 79).

### Taxonomic treatment

#### Gentianella macrosperma

Taxon classificationPlantaeGentianalesGentianaceae

Ma ex H.F. Cao, J.D. Ya & Q.R. Zhang sp. nov.

5FA792F0B5F15255BF64204E4741AF4F

urn:lsid:ipni.org:names:60479356-2

Figures 2 , 3

##### Diagnosis.

Resembles *G. holosteoides*, *G. longicarpa*, *G. sibirica*, *G. stoliczkae* and *G. umbellata*, but differs from them by having even flower size, corolla white, corolla lobe as long as corolla tube, nectaries located close to the throat of the corolla tube and larger seeds.

##### Type.

CHINA. Xinjiang: Ili Kazak Autonomous Prefecture, Gongliu County, Ji’ergelang Township, Qiaxi Village, on the mountain ridge in the forest, 1780 m elev., 6 September 1956, *Shun-Li Chen Tianyi281* (holotype: PE00029466!; isotype: PE00029453!, PE00029471!).

##### Description.

Herbs, annual. Roots slender, yellow. Stems 30–40 cm, erect, subquadrangular, glabrous, yellowish-green, 2.0–2.5 mm in diameter; branched from the base in axils of each node, more slender, suberect or slightly ascending. Leaves opposite, basal leaves not rosette and withered at anthesis, petiole conspicuous, 7–10 mm long, leaves oblong-spatulate, 14–17 × 2–6 mm, base tapering into petiole, margin entire, apex rounded, veins 3–5, raised abaxially and slightly sunken adaxially; lower cauline leaves obovate-spatulate or rounded-spatulate, petiole 10–18 mm long, leaf blades with petiole 18–31 × 10–11 mm, both surfaces glabrous, base tapering into conspicuous petiole, margin entire, apex rounded, veins 5–7 raised abaxially and slightly sunken adaxially; middle leaves on primary stem elliptic, ovate-elliptic, 25–38 × 10–15 mm, base rounded or truncate, inconspicuously short or subsessile, both surfaces glabrous, margin entire, apex rounded, veins 5–9, raised abaxially and slightly sunken adaxially; upper stem leaves ovate-elliptic to ovate, 15–25 × 7–12 mm, with terminal two pairs of leaves nearly in whorls, both surfaces glabrous, base rounded, sessile, margin entire, apex acute, veins 3–5, raised abaxially and sunken adaxially; lateral branches leaves smaller, 10–15 × 4–7 mm. Cymes terminal and axillary, 3–4 flowers per leaf axil, terminal inflorescence 8–10 flowers, dense, inflorescence flowering at different times, pedicel variable in length and up to 36 mm. Flowers 4-merous (rarely 5-merous), all flowers almost the same size (terminal corolla as long as or slightly longer than others), rotating arrangement. Calyx 3.5–4.5 mm long, slightly shorter than corolla or as long as corolla, divided almost to the base, calyx tube 0.7–0.8 mm long, membranous, lobes green, distinctly unequal, 2 slightly larger, oblanceolate to linear-oblanceolate, 3.0–3.5 × 0.7–1.0 mm, 2 (–3) slightly smaller, linear, 2.3–3.0 × 0.4–0.5 mm, apex acute or acuminate, margin scabrous, midvein raised abaxially, sinus obtuse. Corolla white, campanulate, 4.0–4.5(5.0) mm long; corolla tube 2.1–2.4 mm long; lobes ovate, with light brown fine longitudinal veins, 2.2–2.5 × 1.5–1.8 mm, apex obtuse and mucronate, margin entire. Nectaries 8(–10), green, oblong, naked and indistinct, two nectaries per corolla lobe located very close to the throat of the corolla tube, ca. 0.2 mm from the top of corolla tube. Stamens inserted at middle of corolla tube, filaments white, linear, 1.1–1.4 mm long, anthers blue, rectangular, 0.2–0.3 mm long; ovary elliptic, ca. 2.0 mm long. Style short, linear, 0.4–0.5 mm long, stigma small, 2-lobed. Gynophore short, 0.2–0.3 mm long. Capsule elliptic, a concavity sometimes present in the centre, 2.5–4.0 mm long, usually with 2–8 seeds each capsule. Seeds brown, glossy, flat-ellipsoid, 1.2–1.6 × 0.5–0.9 mm, seed coat wrinkled-reticulate (smooth when immature).

**Figure 2.**
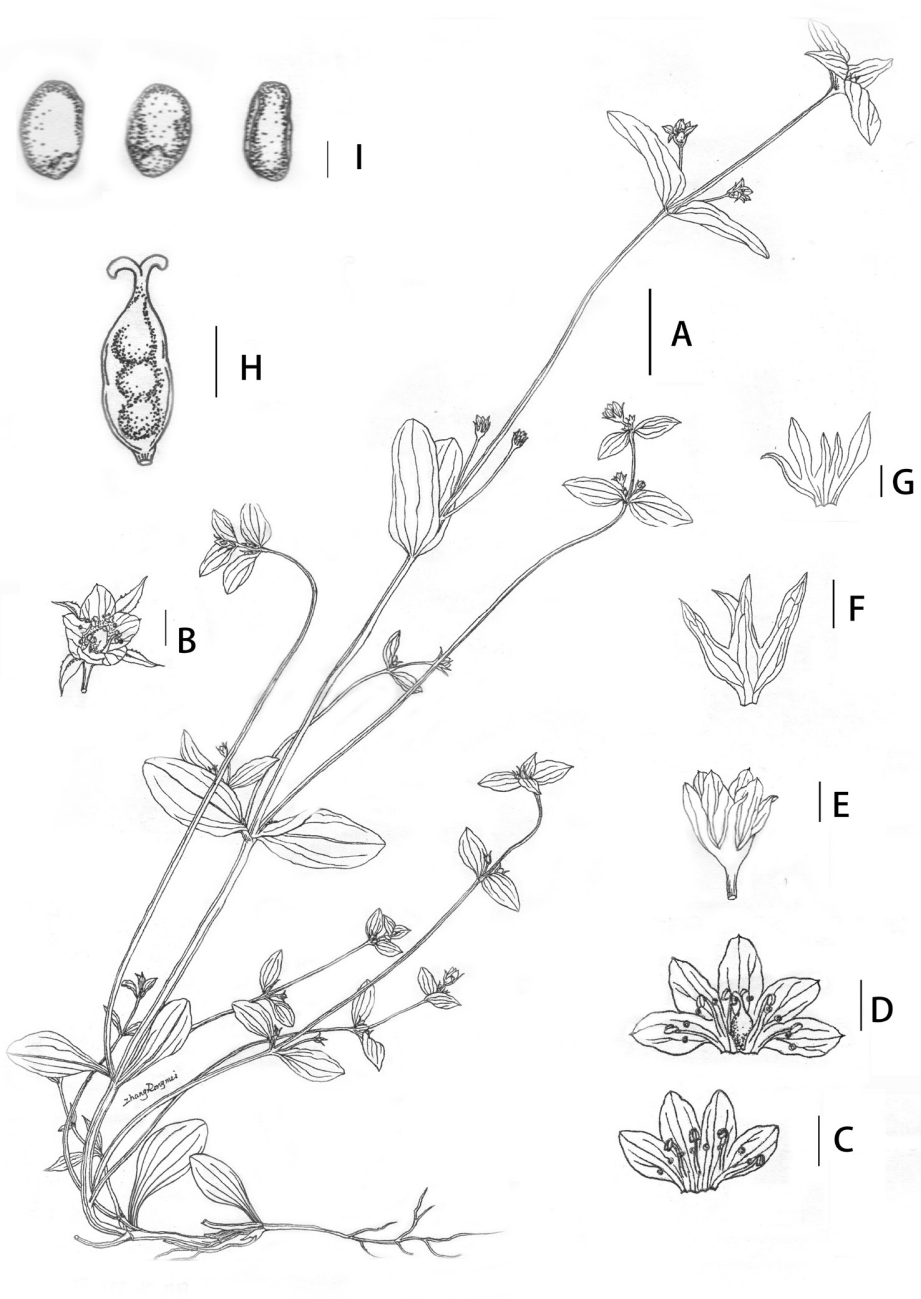
*Gentianella macrosperma*, sp. nov. **A** plant **B** flower, top views **C–D** show opened corollas, 4- and 5-merous, respectively **E** flower, showing the length of calyx and corolla subequal **F** calyx. showing 4-merous **G** calyx, showing 5-merous **H** capsule **I** seeds. Drawn by R.M. Zhang. **H** and **I** from the isotype *S.L. Chen Tianyi281* (PE00029471), others from the paratype *J.D. Ya, Q.R. Zhang & X.J. Hu 17CS16327* (KUN1443565). Scale bars: 2 cm (**A**); 5 mm (**B**); 2 mm (**C–H**); 0.5 mm (**I**).

**Figure 3.**
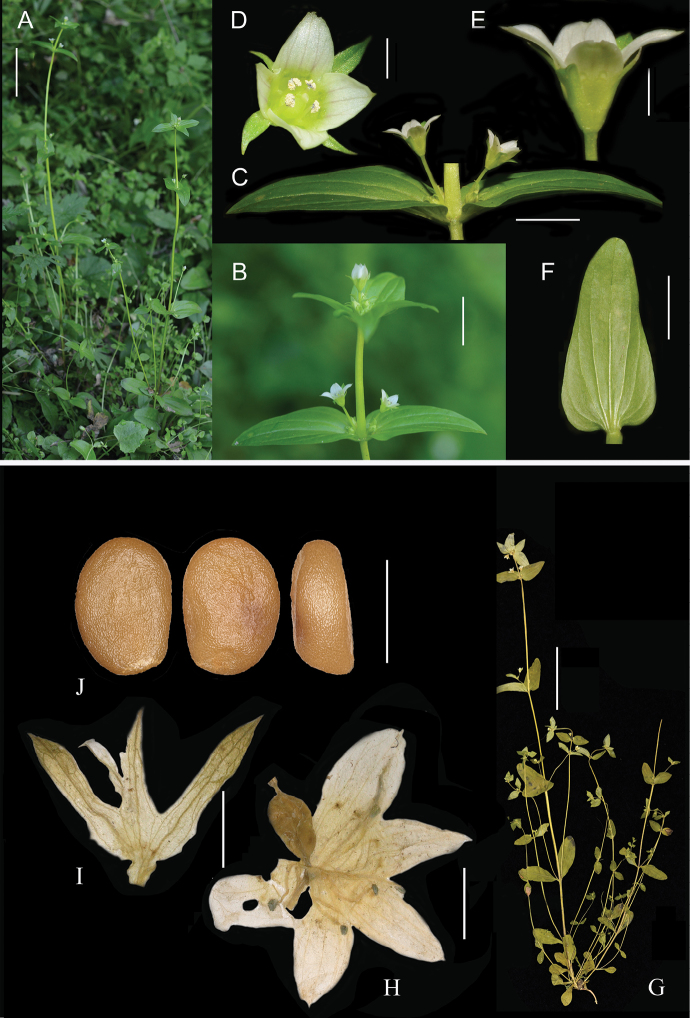
*Gentianella macrosperma*, sp. nov. **A** plant in nature habitat **B** flowers and inflorescence **C** flowers, showing pedicels and upper leaves **D–E** front view and side view of corolla, showing nectaries located close to the throat of the corolla tube **F** middle cauline leaf, abaxial view, showing veins **G** plants specimen (from KUN1443554) **H** opened corolla (5-merous) showing ovary **I** calyx **J** seed, front view (left and middle) and side view (right) (from *S.L. Chen Tianyi281* (PE00029471)). **I**, **H** from the paratype *J.D. Ya, Q.R. Zhang & X.J. Hu 17CS16327* (KUN1443565). Scale bars: 5 cm (**A, G**); 2 cm (**B**); 2 mm (**C–E, I, H**); 1 mm (**J**).

##### Phenology.

Flowering and fruiting from June to September.

##### Distribution and habitat.

*G. macrosperma* is distributed in Gongliu county and Xinyuan county, west of Xinjiang, China. It grows in thickets on the slope or on the mountain ridge in the forest of *Picea schrenkiana* Fisch. & Mey. at an elevation of 1729–1780 m.

##### Etymology.

The specific epithet “macrosperma” refers to the larger seeds of this new species.

##### Vernacular name.

Chinese mandarin: da zi jia long dan (大籽假龙胆)

##### Conservation status.

Currently only known from three localities in west of Xinjiang, therefore considered to be Vulnerable (VU D2) (IUCN 2017).

##### Additional specimens examined (paratypes).

CHINA. Xinjiang: Ili Kazak Autonomous Prefecture, Xinyuan County, on the road from Xinyuan County to the gold mine, 43°16'06.45"N, 83°17'42.90"E, 1729 m elev., 1 July 2017, *J.D. Ya, Q.R. Zhang & X.J. Hu 17CS16327* (KUN1443565!, KUN1443566!, KUN1443554!); Ili Kazak Autonomous Prefecture, Gongliu County, Mohuer Township, Damohe Village, 8 August 1976, *Shu-Run Liu s.n.* (HIMC0026063!, HIMC0026064!. the sheet 0026064 presents a mixture of *Swertia dichotoma* Linn. which was labelled as “A” and *G. macrosperma* labelled as “B”)

## Discussion

It was Prof. Yu-Quan Ma (also as Yu Chuan Ma), a specialist of Gentianaceae, who first recognised this plant as a distinct new species and inscribed the name “*Gentianella macrosperma* Ma” on the specimen kept at PE. Later the same year, he proposed another name “*Gentianella procumbens* Ma” to the same collections, corresponding to its procumbent stems. However, both names were never published. Based on field observation and specimen examination, procumbent stems occurred occasionally in some individuals, the character of larger seeds being easily distinguished from other *Gentianella* species.

In all the known Chinese species of *Gentianella*, the length of corolla lobes is shorter than that of the corolla tube and nectaries which are located at the base or middle of the corolla tube. The same length of corolla lobes and corolla tube and nectaries positioned at the throat of the corolla tube make *G. macrosperma* a distinctive species amongst them. Its large seeds up to 1.6 mm in diameter are perhaps unique amongst the Asiatic species of *Gentianella*.

*G. macrosperma* is similar in size and shape of the corolla lobe to *G. sibirica* and *G. longicarpa*, but further differs from them both in the lack of rosette basal leaves, predominant 4-merous flowers and smaller corolla, no more than 5 mm long, except the corolla lobed to the middle, nectaries position and seeds size. *Gentianella longicarpa*, which is endemic to Afghanistan, is also distinct from *G. macrosperma* in its light-pink, pale blue or lilac-violet flower and larger corolla up to 8 mm long and all calyx lobes are shorter than the corolla tube. *G. macrosperma* is similar in habit and inflorescences to *G. umbellata* and *G. stoliczkae*. The flower of *G. umbellate* is larger than those of *G. macrosperma* and, although the size of the corolla lobe in the two species overlaps, the corolla lobe is much shorter than the corolla tube in *G. umbellate*. In *G. stoliczkae*, flowers are in densely clustered cymes, the corolla are generally much larger up to 20 mm long with various colours from purple, pink, pale blue to yellow and the capsule has a short gynophore ca. 1–2 mm long.

The molecular evidence shows that *G. macrosperma* has the closest relationship with *G. holosteoides* which is native to Turkey and Pakistan and they also share similar floral whorls and basal leaves shape, but plants of *G. holosteoides* are smaller in stature, no more than 5 (7) cm height; it further differs from *G. macrosperma* in its smaller basal leaves, larger flowers with corolla lobes shorter than corolla tube, nectaries position at corolla base and smaller, numerous seeds. A detailed morphological comparison is given in Table 1.

**Table 1.** Morphological comparison between *Gentianella macrosperma* and related species.

	*G. macrosperma*	*G. holosteoides*	*G. longicarpa*	*G. sibirica*	*G. stoliczkae*	*G. umbellata*
Plant height (cm)	12–40	up to 5	9–22	(1–)10–20(–30)	10–45(–60)	(4–)10–35
Basal leaves (mm)	not rosulate, obovate-spathulate 14–17 × 2–6	rosulate, spathulate-ovate or lanceolate, 3–5 × 1–3	rosulate, spathulate, oblong-obovate, 7–16 × 3–8	rosulate, oblong-obovate, 6–20 × 2–6	rosulate, ovate-lanceolate to ovate, 10–35 × 6–20	rosulate, spathulate, obovate-lanceolate, 8–25 × 5–12
Cauline leaves (mm)	ovate to ovaloid, apex rounded, the uppermost sometimes acute, 15–38 × (7–)10–15	lanceolate-oblanceolate or elliptic, apex acute, 5–15 × 2–6	ovate-oblong, ovate or ovate-lanceolate, apex obtuse, the uppermost acute, 8–26 × 4–9	ovate-oblong, ovate-lanceolate, apex acute, 6–20(–35) × 3–9	oblong-lanceolate, lanceolate to ovate-lanceolate, apex acute, (20–)25–40(–50) × (2–)10–15	oblong-ovate, oblong-lanceolate, apex acute, 8–25 × 4–18
Calyx length (mm)	3.5–4.5	4–8	4–5	3–6	8–11	4–10
Floral whorls	4(5)–merous	4(5)–merous	5-merous	5(4)-merous	5-merous	5-merous
Flower size	almost all of the same size	variable in size, terminal ones 1–2 × larger than others	variable in size, terminal ones 1–1.5 × larger than others	variable in size, terminal ones 1–2 × larger than others	variable in size, terminal ones 2–3 × larger than others	variable in size, terminal ones 2–3 × larger than others
Corolla colour	white	pale blue to blue	pale blue, light-pink, or lilac-violet	predominantly pink, yellowish or whitish, rarely pale blue.	purple, pink, pale blue or yellowish	pale azure, purple, pink, yellowish or mixture of these, rarely white
Corolla shape	campanulate	tubular to campanulate-tubular	tubular to campanulate-tubular	tubular or tubular-infundibular	tubular to campanulate-tubular	tubular to campanulate-tubular
Corolla length (mm)	4.0–4.5(–5.0)	6–12	(5–) 6–8	(5–) 6–7 (–10)	7–20	(5–) 8–11 (–15)
Corolla lobes	2.0 mm long, the same length as corolla tube	1.5–3.0 mm long, much shorter than corolla tube	2–3 mm long, shorter than corolla tube	ca. 2 mm long, much shorter than corolla tube	3–7 mm long, much shorter than corolla tube	2–3(4) mm long, much shorter than corolla tube
Nectaries	8(10), at top of corolla tube	8(10), at basal part of corolla tube	10, at basal part of corolla tube	8-10, at basal part of corolla tube	10, at basal part of corolla tube	10, at basal part of corolla tube
Stamens	1.1–1.4 mm	–	–	2–4 mm	ca. 7 mm	1–5 mm
Anthers	blue, 0.2–0.3 mm	–	–	blue, 0.5–0.7 mm	yellow, 1.0–1.2 mm	–
Gynophore	0.2–0.3 mm	subsessile	sessile	subsessile	1.5–2.2 mm	sessile
Seeds	2–8 per capsule, 1.2–1.6 mm in diameter	numerous per capsule, ca. 0.8–1.0 mm in diameter	numerous per capsule, 0.2–0.3 mm in diameter	numerous per capsule, 0.1–0.2 mm in diameter	numerous per capsule, ca. 0.8 mm in diameter	numerous per capsule, 0.2–0.3 mm in diameter

Von Hagen and Kadereit (2001) proposed *Gentianella* s. str. to only include species with one nectary per petal lobe, however, *G. umbellata* and *G. stoliczkae* represented in their study are both binectariate species. Current molecular analyses also shows the binectariate *G. macrosperma* clustered into von Hagen and Kadereit’s *Gentianella* s. str. A careful selection of species across wider geographic regions of this genus and data from more nuclear and chloroplast sequences may clarify the generic circumscription in *Gentianella*.

### Key to species of *Gentianella* in China

The following key is based on Flora of China (Ho and Pringle 1995), Flora of the U.S.S.R. (Shishkin and Bobrov 1967) and other literature (Omer et al. 1988; Aitken 2007; Chen et al. 2011). It includes 11 species of *Gentianella* in China.

**Table:** 

1	Corolla lobes fimbriate at base	***G. acuta***
–	Corolla lobes glabrous at base	**2**
2	Nectaries above the middle of corolla tube	**3**
–	Nectaries at the base of corolla tube	**4**
3	Plant 12–40 cm tall, nectaries close to the throat of corolla tube, seeds 1.2–1.6 mm in diameter	***G. macrosperma***
–	Plant 1–4 cm tall, nectaries just above the middle of corolla tube, seeds 0.7–0.8 mm in diameter	***G. pygmaea***
4	Margin and midvein of calyx lobe blackish	***G. azurea***
–	Calyx not as above	**5**
5	Stem densely purple pilose	***G. gentianoides***
–	Stem glabrous (sometimes sparsely pilose in *G. moorcroftiana*)	**6**
6	Flowers often angled, corolla tube 3–4 times longer than lobe	***G. angustiflora***
–	Flowers not angled, corolla tube 1–3 time(s) longer than lobe	**7**
7	Corolla lobes apically obtuse or round	**8**
–	Corolla lobes apically mucronate	**9**
8	Flowers 5-merous, stem leaf blades linear	***G. moorcroftiana***
–	Flowers 4-merous, stem leaf blades spatulate to oblong-spatulate	***G. arenaria***
9	Corolla lobes densely papillate outside	***G. anomala***
–	Corolla lobes glabrous outside	**10**
10	Corolla 7–20 mm long, terminal ones ca. 20 mm, lobes 3–7 mm	***G. stoliczkae***
–	Corolla 4–10 mm long, terminal ones up to 10 mm, lobes ca. 2 mm	***G. sibirica***

## Supplementary Material

XML Treatment for Gentianella macrosperma

## Appendix

**Appendix 1.** Samples for phylogenetic analysis using matK and ITS sequences with voucher information, GenBank accession number.

Species	Voucher specimen (Herbarium/No.)	Locality	matK	ITS
**OUTGROUP**				
*Chelonanthus alatus* (Aubl.) Pulle	Maas 9316 (U)	French Guiana	KX904551	KX904610
**GENTIANEAE GROUP**				
*Crawfurdia speciosa* Wall.	KEKE 1244 (K)	N/A	AJ010512/AJ011441	AJ294586/AJ294646
*Gentiana crassicaulis* Duthie ex Burkill	xuechy090107 (KUN)	China	KC861277	KC861348
*Gentiana dahurica* Fisch.	xuechy0076 (KUN)	China	KC861279	KC861350
*Gentiana frigida* Haenke	N/A	Germany (Schachen Bot. Garden), cultivated	AJ388166/AJ388236	AJ294588/AJ294648
*Gentiana straminea* Maxim.	xuechy0065 (KUN)	China	KC861282	KC861353
**SWERTIINAE GROUP**				
*Comastoma cyananthiflorum* (Franch.) Holub	XHC120021 (KUN)	China	KC861250	KC861320
*Comastoma cyananthiflorum* (Franch.) Holub	CEE-88 (E 00025334)	China	AJ406324/AJ406353	AJ294585/AJ294645
*Comastoma jigzhiense* T.N. Ho & J.Q. Liu	Chensl0423 (KUN)	China	KC861231	KC861300
*Comastoma polycladum* (Diels & Gilg) T.N. Ho	xuechy090036 (KUN)	China	KC861275	KC861346
*Comastoma pulmonarium* (Turcz.) Toyok.	GLM-081307 (KUN)	China	KC861238	KC861306
*Frasera albicaulis* Griseb.	K. Gutsche 20 (MJG)	N/A	AJ406325/AJ406354	AJ294587/AJ294647
*Gentianella amarella* (L.) Börner	W.J. Schrenk (FR)	N/A	AJ406326/AJ406355	AJ294591/AJ294651
*Gentianella angustiflora* H. Smith	Edinburgh Makalu Expedition 430 (E 00025322)	Nepal	AJ406327/AJ406356	AJ294592/AJ294652
*Gentianella antipoda* (Kirk) T.N. Ho & S.W. Liu	CHR 510015	New Zealand	–	AY136500
*Gentianella arenaria* (Maxim.) T.N. Ho	T.N. Ho et al. 435 (E 00025341)	N/A	AJ406328	AJ294593/AJ294653
*Gentianella aspera* (Hegetschw.) Dostál ex Skalický, Chrtek & Gill	K. Gutsche 45 (MJG)	N/A	AJ010517/AJ011446	AJ294594/AJ294654
*Gentianella astonii* (Petrie) T.N. Ho & S.W. Liu	CHR 509942	New Zealand	–	AY136494
*Gentianella aurea* (L.) H. Smith	H. Smith 4131 (E 00025348)	N/A	AJ406329/AJ406357	AJ294595/AJ294655
*Gentianella auriculata* (Pall.) J.M. Gillett	C. Tpyrgeba (K)	N/A	AJ406330/AJ406358	AJ294596/AJ294656
*Gentianella austriaca* (A. Kern. & Jos.Kern.) Holub	N/A (MJG)	Germany (Schachen Bot. Garden), cultivated	–	AJ294597/AJ294657
*Gentianella azurea* (Bunge) Holub	xuechy090033 (KUN)	China	KC861284	KC861355
*Gentianella azurea* (Bunge) Holub	T.N. Ho, B. Bartholomew, M. Gilbert 1312 (E 00025339)	China	AJ406331/AJ406359	AJ294598/AJ294658
*Gentianella azurea* (Bunge) Holub	Yangyp-Q-0255 (KUN)	China	MN067526*	MK416127*
*Gentianella bellidifolia* (Hook.f.) Holub	19932974	Scotland (Edinburgh Bot. Garden), cultivated	AJ388162/AJ388232	AJ294599/AJ294659
*Gentianella bohemica* Skalický	015	Czech Republic	–	AJ580570
*Gentianella canosoi* G.L. Nesom & B.L. Turner	S. Gonzales, S. Acevedo 2033 (TEX)	N/A	AJ406332/AJ406360	AJ294600/AJ294660
*Gentianella caucasea* (Lodd. ex Sims) Holub	J. C. Archibald 8208 (E 00025347)	N/A	–	AJ294601/AJ294661
*Gentianella cerastioides* (Kunth) Fabris	R. Greissl (MJG)	N/A	AJ010518/AJ011447	AJ294602/AJ294662
*Gentianella cernua* (Kunth) Fabris	C.Viteri 4410 (MO)	N/A	–	AJ294603/AJ294663
*Gentianella cosmantha* (Griseb.) J.S. Pringle	J.G. Haukes, J.P.Hjirting, K. Rahn 3569 (L 424359)	N/A	AJ406333/AJ406361	AJ294604/AJ294664
*Gentianella diemensis* (Griseb.) J.H. Willis	H. Hurka (MJG)	N/A	AJ295332/AJ295333	AJ294605/AJ294665
*Gentianella engadinensis* (Wettst.) Holub	Ge002	Switzerland	–	AJ580559
*Gentianella fastigiata* (Benth.) Fabris	K. Gutsche (MJG)	N/A	–	AJ294606/AJ294666
*Gentianella florida* (Griseb.) Holub	R. Ehrich 444 (MJG)	N/A	AJ406334/AJ406362	AJ294607/AJ294667
*Gentianella foliosa* (Kunth) Fabris	1994-508	England (Kew Bot. Garden), cultivated	–	AJ294608/AJ294668
*Gentianella gentianoides* (Franch.) H. Smith	xuechy090065 (KUN)	China	KC861285	KC861356
*Gentianella gentianoides* (Franch.) H. Smith	xuechy090094 (KUN)	China	KC861286	KC861357
*Gentianella germanica* (Willd.) E.F. Warburg	20	Germany	–	AJ580562
*Gentianella germanica* (Willd.) E.F. Warburg	J.W. Kadereit (MJG)	N/A	AJ406335/AJ406363	AJ294609/AJ294669
*Gentianella hirculus* (Griseb.) Fabris	J.L. Clarke 1787 (QCNE)	N/A	–	AJ294610/AJ294670
*Gentianella holosteoides* Schott & Kotschy ex N.M. Pritch.	Southhampton University 179 (K)	N/A	–	AJ294611/AJ294671
*Gentianella macrosperma* Ma ex H.F. Cao, J.D. Ya & Q.R. Zhang	17CS16327 (KUN)	China	MN067523*	MK416132*
*Gentianella lineata* (Kirk) T.N. Ho & S.W. Liu	CHR 509866	New Zealand	–	AY136503
*Gentianella longicarpa* (Gilli) Holub	D. Podlech 12436 (M)	N/A	–	AJ294612/AJ294672
*Gentianella magellanica* (Gaudich.) Fabris	K. Kubitzki, T. Feuerer 99-10 (MJG)	N/A	AJ406336/AJ406364	AJ294613/AJ294673
*Gentianella microcalyx* (Lemmon) J. M. Gillett	E. Joyal, J. Enrique 1853 (TEX)	N/A	AJ406337/AJ406365	AJ294614/AJ294674
*Gentianella montana* (G. Forst.) Holub	CHR 509944	New Zealand	–	AY136491
*Gentianella moorcroftiana* (Wall. ex G. Don) A. Shaw	R. McBeath 2093 (E 00025318)	N/A	AJ406338/AJ406366	AJ294615/AJ294675
*Gentianella narcissoides* (Gilg) T.N. Ho & S.W. Liu	L. Naessany 14 (MJG)	N/A	–	AJ294616/AJ294676
*Gentianella patula* (Kirk) Holub	19932978	Scotland (Edinburgh Bot. Garden), cultivated	AJ406339/AJ406367	AJ294617/AJ294677
*Gentianella peruviana* (Griseb.) Fabris	19950534	Scotland (Edinburgh Bot. Garden), cultivated	AJ388163/AJ388233	AJ294618/AJ294678
*Gentianella propinqua* (Richardson) J.M. Gillett	G. Halliday A 333/75 (E 00025300)	North America	AJ406340/AJ406368	AJ294619/AJ294679
*Gentianella quinquefolia* (L.) Small	Bozeman, Ramseur, Radford 45200 (E 00025241)	North America	AJ406341/AJ406369	AJ294620/AJ294680
*Gentianella quinquefolia* (L.) Small	D. Pittillo 12106 (WCUH)	America	–	EU812469
*Gentianella rapunculoides* (Willd. ex Schult.) J.S. Pringle	R. Greissl 616 (MJG)	N/A	–	AJ294621/AJ294681
*Gentianella ruizii* (Griseb.) Holub	Weigend, Weigend 2000/386 (NY)	N/A	AJ406342/AJ406370	AJ294622/AJ294682
*Gentianella rupicola* (Kunth) Holub	199930516	Scotland (Edinburgh Bot. Garden), cultivated	–	AJ294623/AJ294683
*Gentianella saxosa* (G. Forst.) Holub	Gutsche (MJG)	N/A	–	AJ406343/AJ406371
*Gentianella splendens* (Gilg) Fabris	J.L. Clarke 1855 (QCNE)	N/A	AJ295336/AJ295337	AJ294624/AJ294684
*Gentianella stoliczkae* (Kurz ex C.B. Clarke) Holub	LiuJQ0028 (KUN)	China	MN067524*	MK416130*
*Gentianella stoliczkae* (Kurz ex C.B. Clarke) Holub	LiuJQ0071 (KUN)	China	MN067525*	MK416131*
*Gentianella stoliczkae* (Kurz ex C.B. Clarke) Holub	O. Anders 8178 (M 50043)	N/A	AJ406344/AJ406372	AJ294625/AJ294685
*Gentianella sulphurea* (Gilg) Fabris	J.L. Clarke 1833 (QCNE)	N/A	–	AJ294626/AJ294686
*Gentianella thyrsoidea* (Hook. f.) Fabris	D.N. Smith, F. Escalona 10134 (MO)	N/A	–	AJ294627/AJ294687
*Gentianella tristicha* (Gilg) Fabris ex T.N. Ho & S.W. Liu	D.N. Smith, F. Escalona 10125 (MO)	N/A	–	AJ294628/AJ294688
*Gentianella umbellata* (M. Bieb.) Holub	K91-G3	Georgia	–	Z48102/Z48132
*Gentianella wislizenii* (Engelm.) J.M. Gillett	M. Lavin 4947 (TEX)	N/A	–	AJ294630/AJ294690
*Gentianopsis barbata* (Froel.) Ma	xuechy090085 (KUN)	China	KC861287	KC861358
*Gentianopsis crinita* (Froel.) Ma	N/A (MJG)	Germany (Mainz Bot. Garden), cultivated	AJ406345/AJ406373	AJ294631/AJ294691
*Halenia elliptica* D. Don	GLM-081543 (KUN)	China	KC861242	KC861310
*Halenia palmeri* A. Gray	K.B.v. Hagen 98/41 (MJG)	N/A	-	AJ294632/AJ294692
*Jaeschkea oligosperma* (Griseb.) Knobl.	R. McBeath 2300 (E 00025275)	N/A	AJ388171/AJ388241	AJ294633/AJ294693
*Lomatogonium bellum* (Hemsl.) H. Smith	GLM-06075 (KUN)	China	KC861237	KC861305
*Lomatogonium carinthiacum* (Wulfen) Rchb.	V. Zuev 6649 (BR)	N/A	AJ406346/AJ406374	AJ294634/AJ294694
*Lomatogonium forrestii* (I.B. Balfour) Fernald	XHC120061 (KUN)	China	KC861261	KC861332
*Lomatogonium gamosepalum* (Burkill) H. Smith	GLM-081372 (KUN 1272996)	China	KC861241	KC861309
*Lomatogonium oreocharis* (Diels) C. Marquand	CLD-90 1106 (K)	N/A	AJ388174/AJ388244	AJ294635/AJ294695
*Megacodon stylophorus* (C.B. Clarke) H. Smith	GLM-081957 (KUN)	China	KC861245	KC861313
*Megacodon stylophorus* (C.B. Clarke) H. Smith	Kuming, Edinburgh, Gothenburgh Exp. 1378 (E 00025279)	China	AJ388177/AJ388247	AJ294636/AJ294696
*Swertia bifolia* Batalin	Chensl0388 (KUN)	China	KC861229	KC861298
*Swertia bimaculata* (Sieb. & Zucc.) Hook. f. & Thomson ex C.B. Clarke	XHC120026 (KUN)	China	KC861264	KC861335
*Swertia bimaculata* (Sieb. & Zucc.) Hook. f. & Thomson ex C.B. Clarke	XCY090050 (KUN)	China	JF956557	JF978820
*Swertia cincta* Burkill	XCY090098 (KUN)	China	JF956561	JF978823
*Swertia crassiuscula* Gilg	U. Hecker 1094 (MJG)	N/A	AJ406347/AJ406375	AJ294637/AJ294697
*Swertia decora* Franch.	XCY090077 (KUN)	China	JF956567	JF978825
*Swertia erythrosticta* Maxim.	xuechy090044 (KUN)	China	KC861267	KC861338
*Swertia franchetiana* H. Smith	XHC120048 (KUN)	China	KC861256	KC861326
*Swertia japonica* (Schult.) Makino	N/A (KYO)	Japan (Kyoto Bot. Garden), cultivated	AJ406348/AJ406376	AJ294638/AJ294698
*Swertia macrosperma* (C.B. Clarke) C.B. Clarke	XHC120060 (KUN)	China	KC861260	KC861331
*Swertia macrosperma* (C.B. Clarke) C.B. Clarke	J.H. de Haas 2765 (U 500099)	N/A	AJ406349/AJ406377	AJ294639/AJ294699
*Swertia nervosa* (G. Don) Wall. ex C.B. Clarke	XHC120053 (KUN)	China	KC861258	KC861328
*Swertia patens* Burkill	09CS1123 (KIB)	China	KC861233	KC861302
*Swertia perennis* L.	K.B. Hungerer (MJG)	N/A	–	AJ294640/AJ294700
*Swertia punicea* Hemsl.	19943574	Scotland (Edinburgh Bot. Garden), cultivated	AJ406350/AJ406378	AJ294641/AJ294701
*Swertia racemosa* (Wall. ex Griseb.) C.B. Clarke	J.H. de Haas 2725 (U 500131)	N/A	AJ406351/AJ406379	AJ294642/AJ294702
*Swertia volkensii* Gilg	U. Hecker 1093 (MJG)	N/A	AJ406352/AJ406380	AJ294643/AJ294703
*Swertia yunnanensis* Burkill	XCY090089 (KUN)	China	JF956585	JF978836
*Veratrilla baillonii* Franch.	Kuming, Edinburgh, Gothenburgh Exp. 1326 (E 00025273)	China	AJ388196/AJ388266	AJ294644/AJ294704

* indicates the taxon was newly sequenced in the present study.
